# Geotechnical investigation of soil properties in Hatsalatladi village, Botswana; Insights from aeromagnetic, laboratory soil tests and Rayleigh wave dispersion datasets

**DOI:** 10.1016/j.dib.2023.109345

**Published:** 2023-07-04

**Authors:** Olorato Mosweu, Moikwathai Moidaki, Rubeni T. Ranganai, Lucky C. Moffat, Rapelang E. Simon, Kebabonye Laletsang, James G. King, Nata T. Tafesse, Reneilwe Lasarwe, Motsamai Kwadiba, Mpho Ramaselaga, Mooketsi Vike

**Affiliations:** a Department of Physics, Faculty of Science, University of Botswana, Private Bag UB00704, Gaborone, Botswana; b Department of Geology, Faculty of Science, University of Botswana, Private Bag UB00704, Gaborone, Botswana; c Botswana Geoscience Institute, Private Bag 0014, Lobatse.3, Botswana

**Keywords:** Hatsalatladi, Ground fissures, MASW, Soil plasticity, Shear wave velocity

## Abstract

Soil tests and Multichannel Analysis of Surface Waves (MASW) data were conducted in Hatsalatladi village, Botswana, to investigate the occurrence of ground fissures within the village and to identify the likely causes of the fissures and their depth extent. The MASW data were collected to gain insights into the variation of shear wave velocity with depth. The dataset shows that the shear wave velocity ranged from 150 m/s – 500 m/s, with Poisson's ratios ranging from 0.02 to 0.25. A low-velocity zone (LVZ) was observed in the upper 5 m of the subsurface with velocities ranging from 200 m/s to 350 m/s.

The soil plasticity was measured through the plastic and liquid Atterberg tests. Atterberg limits measurements obtained from the three survey sites show that the plastic index of the soil samples collected from depths of 1 m fall within the 10–20% range. Specifically, the Filled Crack survey site had a plastic index of 16%, while the Abandoned House and Bridge sites had 18.7% and 13.5%, respectively. Soil samples from Filled Crack and Abandoned House site revealed a linear shrinkage of 6.4%, while the Bridge site soil sample had a linear shrinkage of 2.9%. The sieve analysis test results are also presented.

**Specifications Table**

**Table:** 

Subject	Earth and Planetary Sciences

Specific subject area	Geophysics: datasets provide insights on geology and geotechnical aspects on the processes that lead to the development of pervasive ground fissures.
Type of data	Table Graph Figure
How the data were acquired	A grid of high-resolution aeromagnetic data was obtained from the Botswana Geoscience Institute. The Oasis Montaj software package was used for gridding and filtering the magnetic data. Seismic data (MASW: Multichannel Analysis of Surface Waves) were collected in Hatsalatladi village using a 24 channel Geometrics data logger with geophones spaced at 2 m intervals with an offset of 4 m. Multiple spread data were acquired at each survey site. The SurfSeis version 3 software from the Kansas Geological Survey was used to process the MASW data and analyze the dispersive characteristics of Rayleigh waves. Soil samples for geotechnical investigations were collected at survey sites at an average depth of 1 m.
Data format	Raw seg2 seismic data Analyzed Filtered
Description of data collection	Gridded magnetic data were obtained from the Botswana Geoscience Institute. MASW was used to approximate the shear-wave velocity with depth by utilizing the dispersive properties of Rayleigh waves. Elastic waves were produced by a sledgehammer, and a 24-channel seismograph was used for data acquisition with geophones equally spaced at 2 m intervals with an offset of 4 m. The spread was then shifted by 4 m until the whole profile was covered. Soil tests involved sieve analysis, shrinkage limit, and liquid and plastic limit measurements. Soil samples that were collected were placed in labelled plastic bags for further tests in the laboratory.
Data source location	The datasets used in this work were collected in Hatsalatladi village, located at latitude 24.13˚S and longitude 25.59˚E, 40 km north of Molepolole Village in Botswana. The three profiles for MASW data acquisition are as follows: The coordinates of the first profile at the beginning and end were recorded as -24.14101°S 25.58724°E and -24.14109°S 25.58643°E, respectively. The second survey point was a fissure which goes across a house (pink house), and in the oriented SE-NW direction. This crack made the occupants to vacate the house due to compromised structural integrity. The coordinates of the start and end of the second profile were recorded as -24.13058°S 25.59180°E and -24.12964°S 25.59137°E, respectively. The last profile was situated on a fissure near the Bridge/tarred road. The ground crack stretches for more than 500 m towards Botlhapatlou village and also branches into a stream towards its endpoint. The survey line coordinates at the start and end of the profile were recorded as -24.12988°S 25.59150°E and -24.12992°S 25.58883°E, respectively. The average elevation of the three profiles was about 1040 ± 10m.
Data accessibility	Repository name: Mendeley Data repository Data identification number: DOI: 10.17632/2dvz4mfmw9.1 Direct URL to data: https://data.mendeley.com/datasets/2dvz4mfmw9/1 **Instructions to open the different data file formats:** .grd is grid file generated by Geosoft Oasis Montaj software. It can be opened by Geosoft Viewer from: https://www.seequent.com/products-solutions/geosoft-viewer/.
	LST: files ending with .LST are text files that contain information about the dispersion points picked on the fundamental mode. They can be opened by any text editing software e.g. notepad and WordPad The .LST files were generated by SurfSeis software during the calculation of 1D variation of shear-waves with depth and the information about Frequency, initial and final shear wave velocities and RMSE are provided as well as number of iterations. Other software packages for analysing surface waves might have a different file structure. All the files ending with .IVO, .DC, .DCT, and .IND are text files and were generated by SurfSeis during data processing. They can be opened by and text editor such as Notepad and WordPad. Files ending with .JPG are images (jpeg images) and can be opened by any image editing software like Paint or Corel draw. .sg2: The .sg2 format is a binary file format used for storing seismic data. .sg2 file contains seismic data in the form of time series, which are sampled at regular intervals. To work with .sg2 files, specialized software tools are required such as Seismic Unix which is a popular software package for processing and analyzing seismic data in the .sg2 format. Other software tools that support .sg2 files include ProMAX, GeoFrame, Kingdom and SurSeis. SurfSeis Vesrion 3 developed by Kansas Geological Survey in the United States of America was used in this work.

## Value of the Data

• Utilize Rayleigh wave dispersion characteristics to improve understanding of soil stiffness in the vadose region of Hatsalatladi, using new MASW and geotechnical data.
• Analyze aeromagnetic data to determine the location of Hatsalatladi village in relation to geological terrains and provide insights for future research.
• Identify low-velocity zones to help local inhabitants avoid areas susceptible to ground cracking and other hazards.
• Provide valuable data for the Botswana government to support major structural developments and inform decision-making.
• Support other government sectors, such as tarred road construction, village electrification, and sewage disposal, by providing a comprehensive dataset for policy makers to use.

## 1 Objective

Hatsalatladi village, located 30 km north of Molepolole along Shoshong road, has been experiencing intense ground fissuring over the years, which has become a national concern. Despite the ground fissures, the settlement has grown to become a village with modern infrastructure, such as a clinic, library, community hall, primary school, and tarred road. Unfortunately, both government and public infrastructure have been affected, as the local government had to demolish two classroom blocks in a primary school after their damage became a hazard to the pupils. Additionally, some residents had to relocate from their homes as their houses were no longer safe. These ground cracking incidences necessitated a need for research to document the probable cause, lateral extent, and depth of ground fissures. The objective of this research is to identify the potential cause, extent, and depth of ground fissures in Hatsalatladi village. This will be achieved by using Multichannel Analysis of Surface Waves (MASW) data and soil tests to analyze the characteristics of the soil, including Atterberg limits and sieve analysis. Additionally, Total Magnetic Intensity (TMI) data will be used to gain insights into subsurface geologic structures. The results obtained from this research will be useful for identifying areas that are prone to ground cracking and providing guidance for government-funded structural developments, tarred road construction, village electrification, and sewage disposal.

## 2 Data Description

**Fig. 1:**
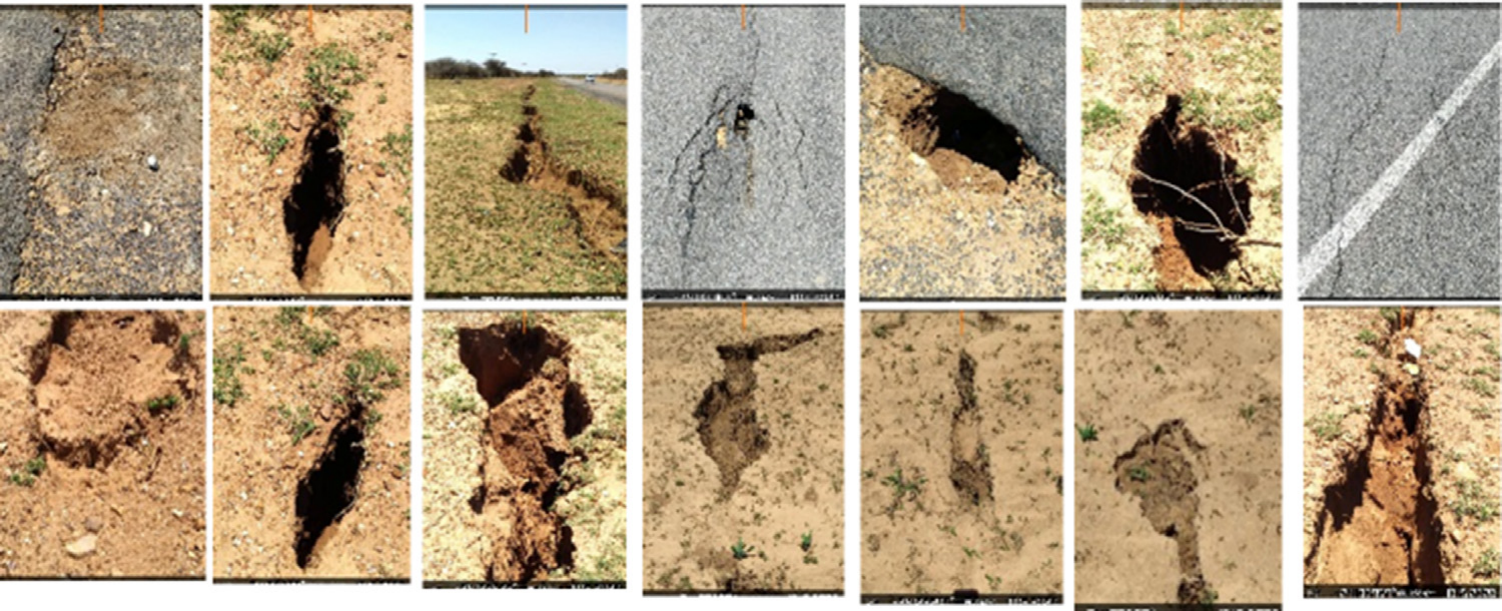
Ground fissures in Hatsalatladi Village. The fissures/cracks are randomly distributed all over the village with a N-S and NE-SW orientation. Measured widths ranges from a few millimeters to 2 meters in some places and the depth ranges from a few centimeters to 1.5 m.

**Fig. 2:**
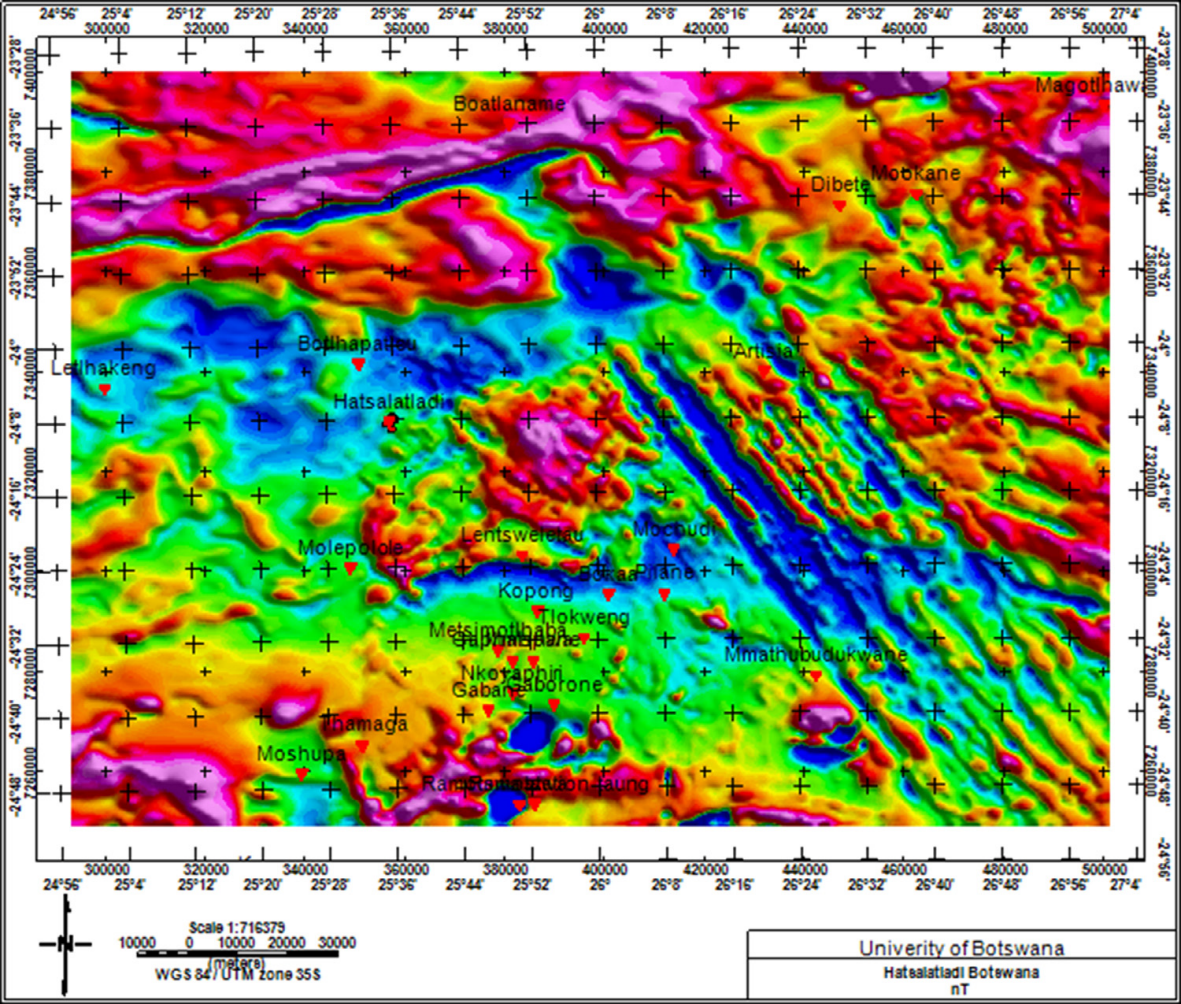
Geographic location of Hatsalatladi on Total magnetic Intensity map.

**Fig. 3:**
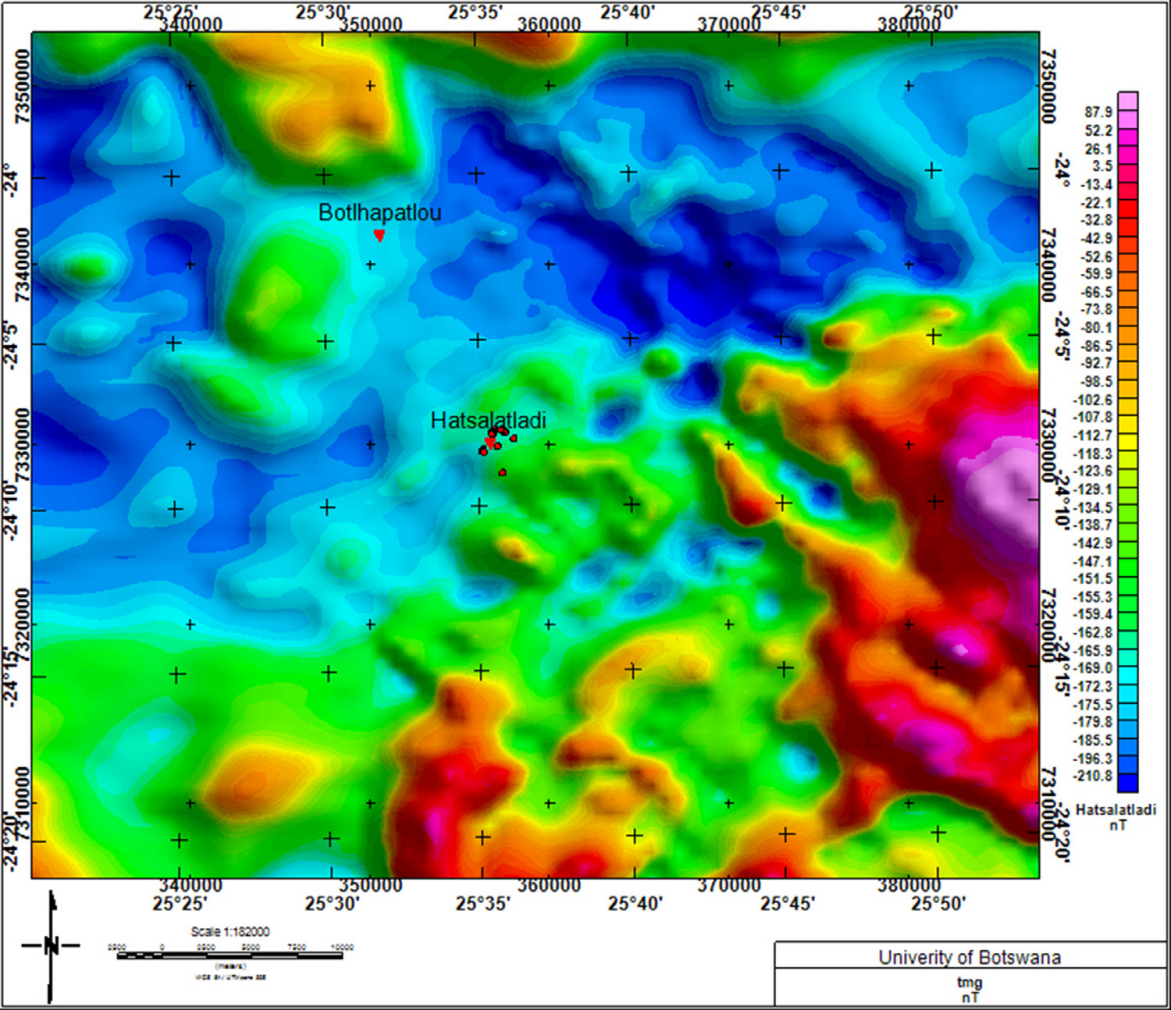
Total Magnetic Intensity (TMI) map showing Hatsalatladi on an area with low magnetic intensity. Coordinates of ground fissures (Table 1: represented by red dots) are plotted on the TMI map. The legend indicates that, Hatsalatladi village is located within a low magnetic susceptibility region (within the blue and green legend colors).

The dataset used in this research is stored in the Mendeley data repository [1] and organized into three subdirectories under Research_data-Hatsalatladi. The three subdirectories are named: (1) Magnetic dataset, (2) MASW dataset, and (3) Soil test dataset. The aeromagnetic data used in this work was processed with Geosoft Oasis Montaj software [2], where the International Geomagnetic Reference Field (IGRF) model of the core field was subtracted from the observed total field to obtain the residual total field. The minimum curvature technique [3] was used for gridding with a grid cell size of 62.5 m [4]. The grid was re-projected to WGS84 and UTM35S. The mapped fissures observed on the surface in Hatsalatladi are shown in Fig. 1, and their geographic locations and azimuths are tabulated in Table 1 and plotted in. Fig. 2. Fig. 3 shows a zoomed-in section of Hatsalatladi village with underlying geologic structures such as contacts and low-magnetized lineaments.

**Table 1:** location of ground crack and their orientation.

Longitude	Latitude	elevation (m)	Direction	azimuth
25.585767	-24.139157	1127.2	NW	305
25.585773	-24.139151	1114.8	N	352
25.585795	-24.13912	1117.2	E	80
25.585828	-24.139143	1108.7	SE	123
25.585676	-24.139496	1121.9	S	181
25.585751	-24.139166	1110.7	N	343
25.585885	-24.139286	1106.8	E	79
25.585847	-24.1395	1108.1	W	260
25.596335	-24.150182	1119.1	E	95
25.593897	-24.13687	1105.7	SW	218
25.602559	-24.133028	1097.2	SE	128
25.597924	-24.129737	1109	E	101
25.597943	-24.12973	1104.4	E	95
25.597157	-24.129449	1110.5	N	22
25.596124	-24.12837	1107.6	SE	147
25.596023	-24.128277	1106	SE	115
25.596018	-24.128277	1105.8	E	85
25.596017	-24.128273	1108.5	SE	148
25.396041	-24.128235	1108.2	NW	331
25.595756	-24.128433	1105.7	N	355
25.592707	-24.128489	1108.1	N	10
25.592707	-24.128489	1108.1	N	10
25.592707	-24.128489	1108.1	N	10
25.592708	-24.128497	1108.3	SW	226
25.592402	-24.128411	1110.5	W	290
25.590887	-24.129407	1108.8	N	8
25.591277	-24.13002	1111.4	NE	63
25.591364	-24.129937	1112.2	SW	242
25.591432	-24.129866	1111.7	NE	47
25.591663	-24.129789	1110.8	SE	127
25.591836	-24.129902	1111	SE	156
25.591794	-24.129958	1111.9	S	181
25.591709	-24.130059	1111.2	W	257
25.591636	-24.130108	1111	N	23
25.591588	-24.130218	1110.5	E	107
25.591573	-24.130271	1112.5	S	167
25.59158	-24.130339	1111.5	NE	58
25.591593	-24.130324	1111.1	NE	58
25.591538	-24.130419	1109.5	SE	122
25.590765	-24.130749	1111.7	SW	234
25.58584	-24.138022	1117.5	N	13
25.585839	-24.138192	1118.6	S	177
25.585827	-24.138393	1117.7	W	275
25.585832	-24.138371	1117	N	9
25.585794	-24.138464	1117.2	NW	301
25.585794	-24.138571	1118.5	S	183
25.585777	-24.138612	1118.3	S	194
25.585779	-24.138629	1117.7	NE	25
25.585779	-24.138649	1117.8	SW	226
25.585788	-24.138674	1118.8	N	343
25.585789	-24.138677	1117.9	SE	143
25.585778	-24.138757	1119.1	N	352
25.585772	-24.138872	1119.1	SW	241
25.585767	-24.138993	1118.2	E	77
25.585751	-24.139029	1119	N	6
25.585768	-24.139118	1118.3	SW	213
25.585762	-24.139178	1117.9	S	161
25.585775	-24.139211	1117.4	NW	310
25.585912	-24.139278	1119.3	SW	230
25.585912	-24.139262	1119.5	S	173
25.585808	-24.139307	1118.7	SE	173

**Fig. 4:**
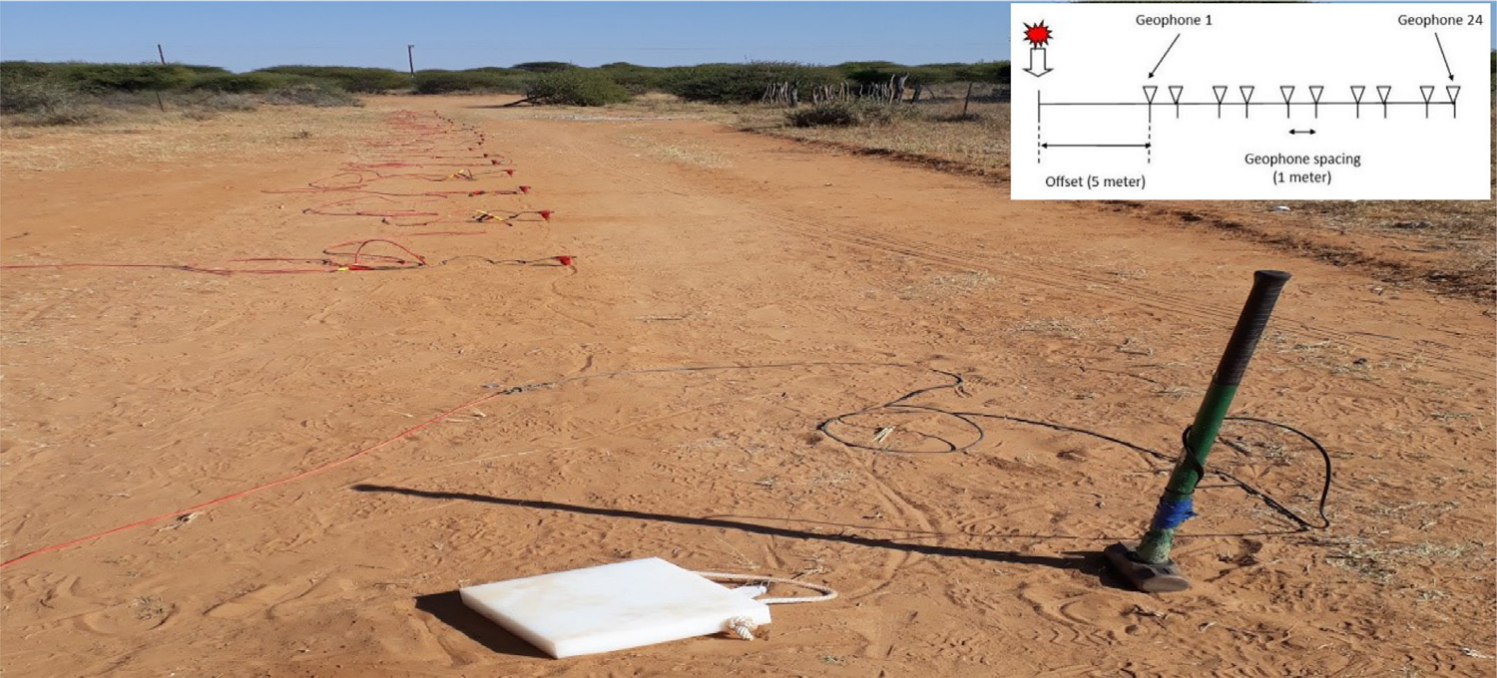
Field layout of Geometrics-24 channel seismograph showing geophone spacing and an offset. Equipment set up of the MASW profile (Filled Crack profile). The coordinates of the start and end of the survey line are -24.1410085S, 25.5872383E and -24.1410867S, 25.586429E respectively.

**Fig. 5:**
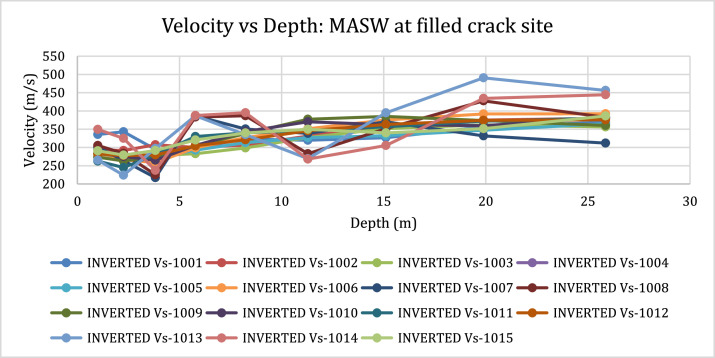
1D-variation of shear wave velocity with depth at the Filled Crack site using a 10 layer model.

**Fig. 6:**
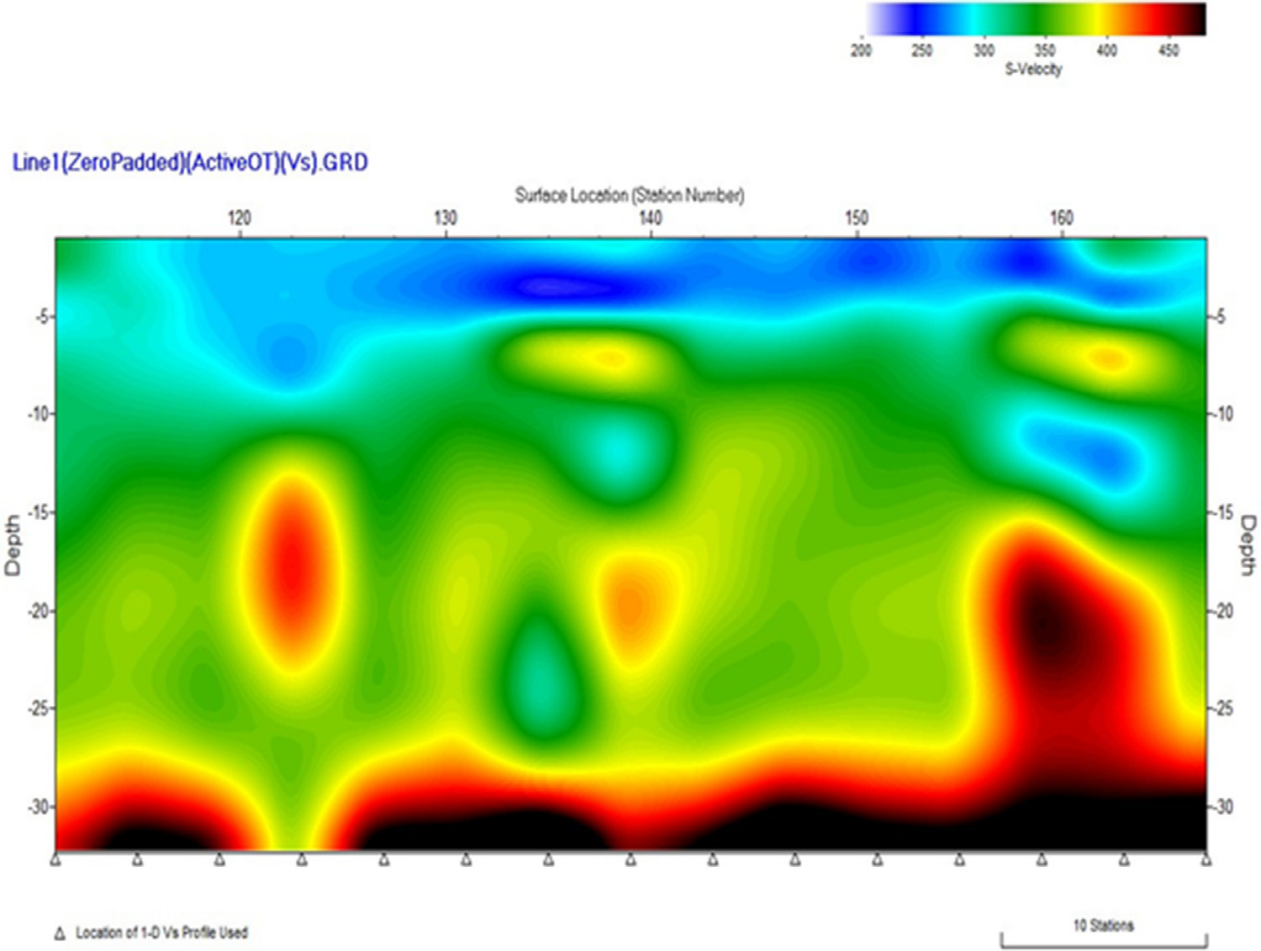
2-D view of shear-wave velocity with depth at the Filled Crack site. The pseudo section shows a saturated low velocity layer (LVL) represented in blue up to a depth of 6 m underlain by weathered basement dolomites. A layer with high shear wave velocities is encountered at a depth of 15 m.

**Fig. 7:**
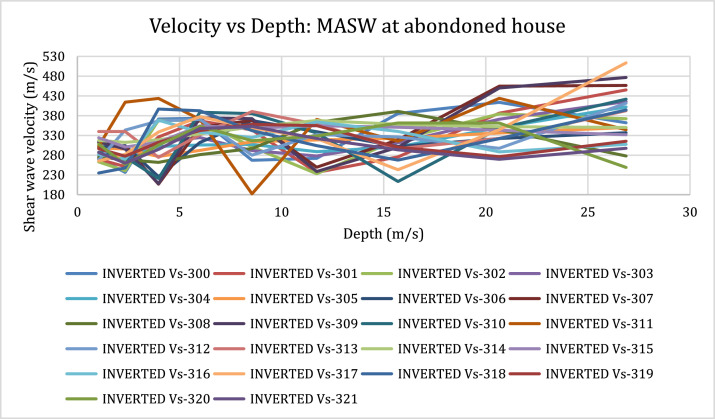
1D-variation of shear wave velocity with depth at Abandoned House site using a 10-layer model.

**Fig. 8:**
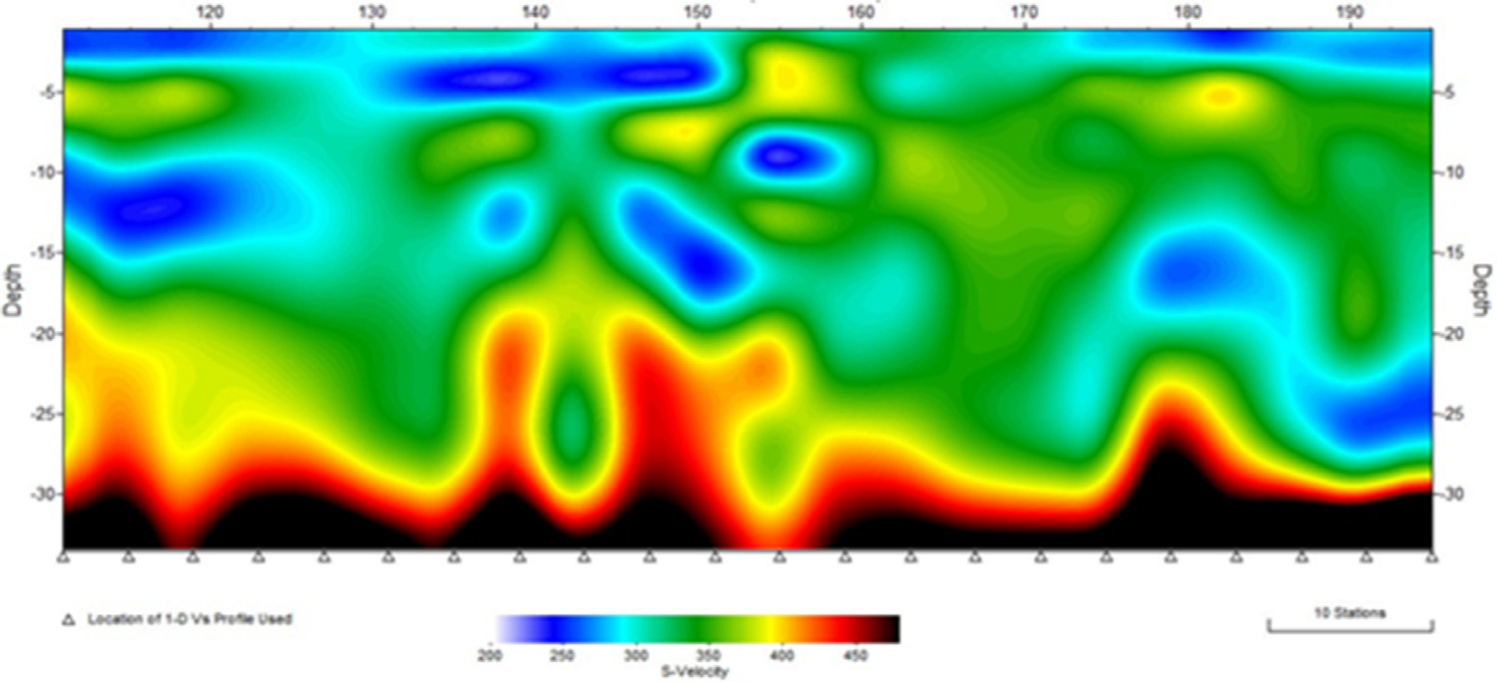
2-D view of shear-wave velocity with depth at the Abandoned House. 2-D Velocity model at the Abandoned House survey site. The pseudo section shows a saturated low velocity layer (blue zones) in the range of 2 m to 5 m thick underlain by pockets of LVZ within the weathered basement dolomites. A layer with high shear wave velocities is encountered at a depth of 20 m.

**Fig. 9:**
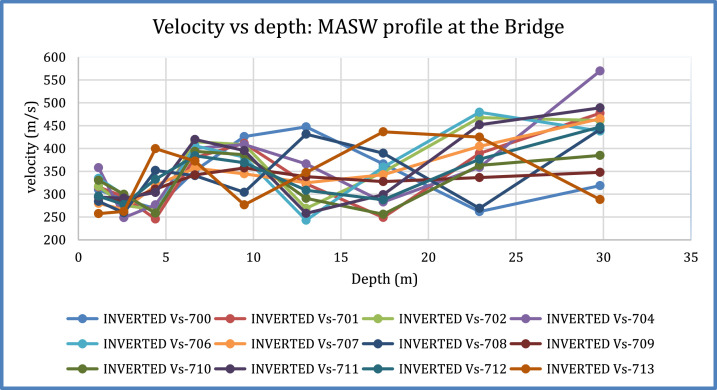
1D-variation of shear wave velocity with depth at Abandoned House site using a 10 layer model.

**Fig. 10:**
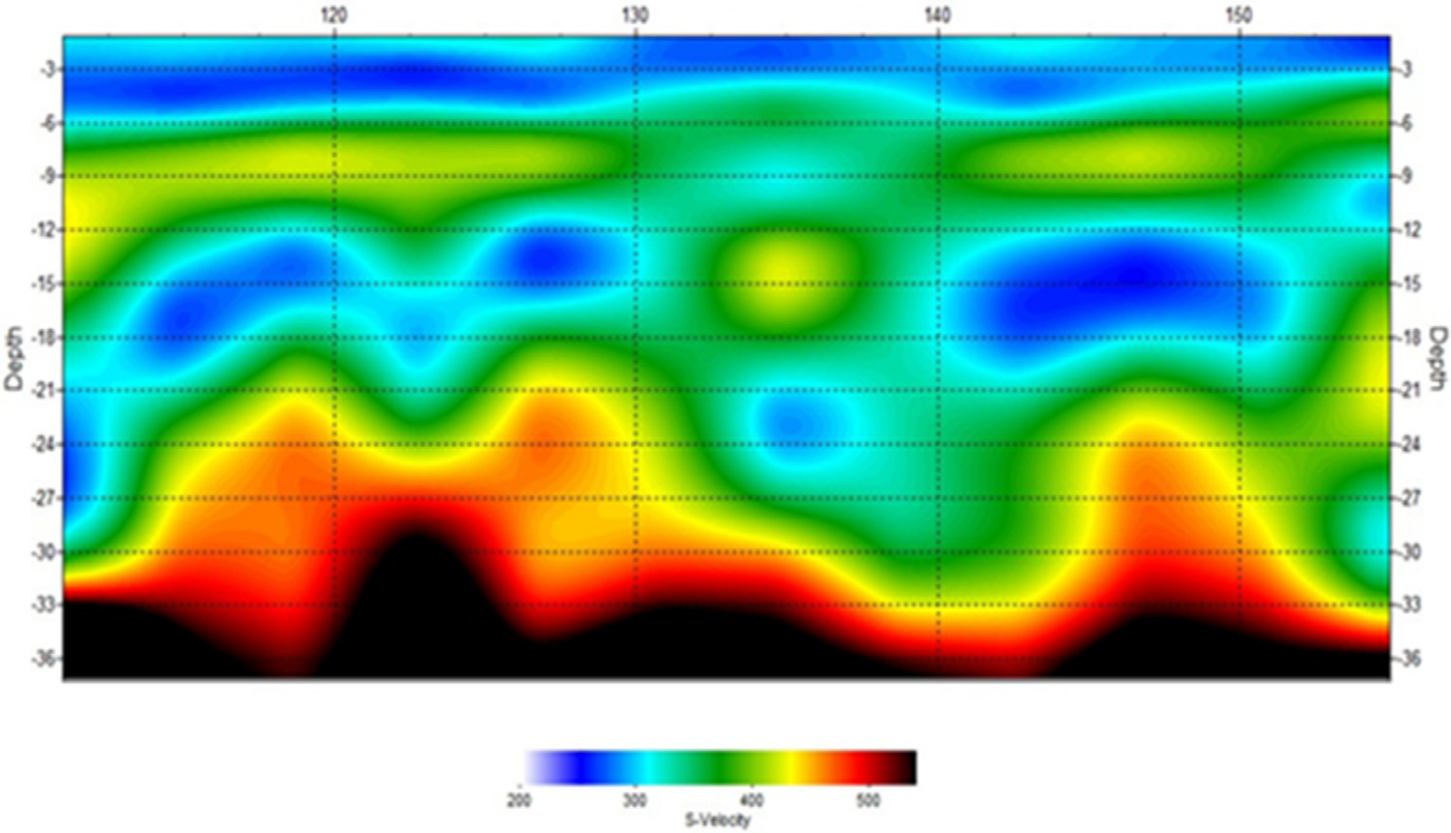
2-D Velocity model at the Bridge survey site. The pseudo section shows a saturated low velocity layer of 4 m thick underlain by weathered dolomites. Within this weathered layer of dolomites there is another discontinuous layer of low velocity.

The MASW dataset subdirectories has 6 subdirectors. MASW seg2 data files and dispersion data are stored in Abandoned_House_seg2_data, Bridge site_seg2_data and Filled crack_seg2_data for Abandoned House site, Bridge site and Filled Crack site respectively. Included with these subdirectories is an excel file named Hatsalatladi_MASW-excel graphs which has all the dispersion figure used in this work. The MASW profiles were run perpendicular to the strike of selected fissured areas, with the following field configurations: an offset distance of 4 m and a geophone spacing of 2 m. The sampling rate and record length were set at 0.25 ms and 1 ms, respectively. The spread was moved by 4 m until the whole survey line was covered, involving moving the first two geophones to the end of the spread line, and three stacks were summed up per shot (Fig. 4). The MASW method utilizes the dispersion property of surface waves for Vs profiling in 1D (depth) or 2D (depth and surface location) format [5,6]. It is essentially an engineering seismic method that deals with frequencies ranging from 3-30 Hz, recorded by a multichannel (24 or more channels) seismograph with an array of geophones deployed along the profile. The MASW method actively generates surface waves (Rayleigh waves) through an impact source like a sledgehammer. MASW data is acquired in seg2 format, which is accepted by SurfSeis from the Kansas Geological Survey. The processing of MASW data involves three steps, as outlined by [5]: (1) acquiring multichannel field records (or shot gathers); (2) extracting dispersion curves (one from each record); and (3) inverting these dispersion curves to obtain 1D (depth) VS profiles (one profile from one curve). By placing each 1D Vs profile at a surface location corresponding to the middle of the receiver line, a 2D (surface and depth) Vs map can be constructed through an appropriate interpolation scheme (Fig. 1 1). The MASW processing outputs are shown in Figs. 5, 7, and 9 for the 1D variation of shear wave velocity with depth, and Figs. 6, 8, and 10, which show the 2D variation of shear-wave velocity with depth for the Filled Crack site, Bridge site, and Abandoned House site, respectively.

**Fig. 11:**
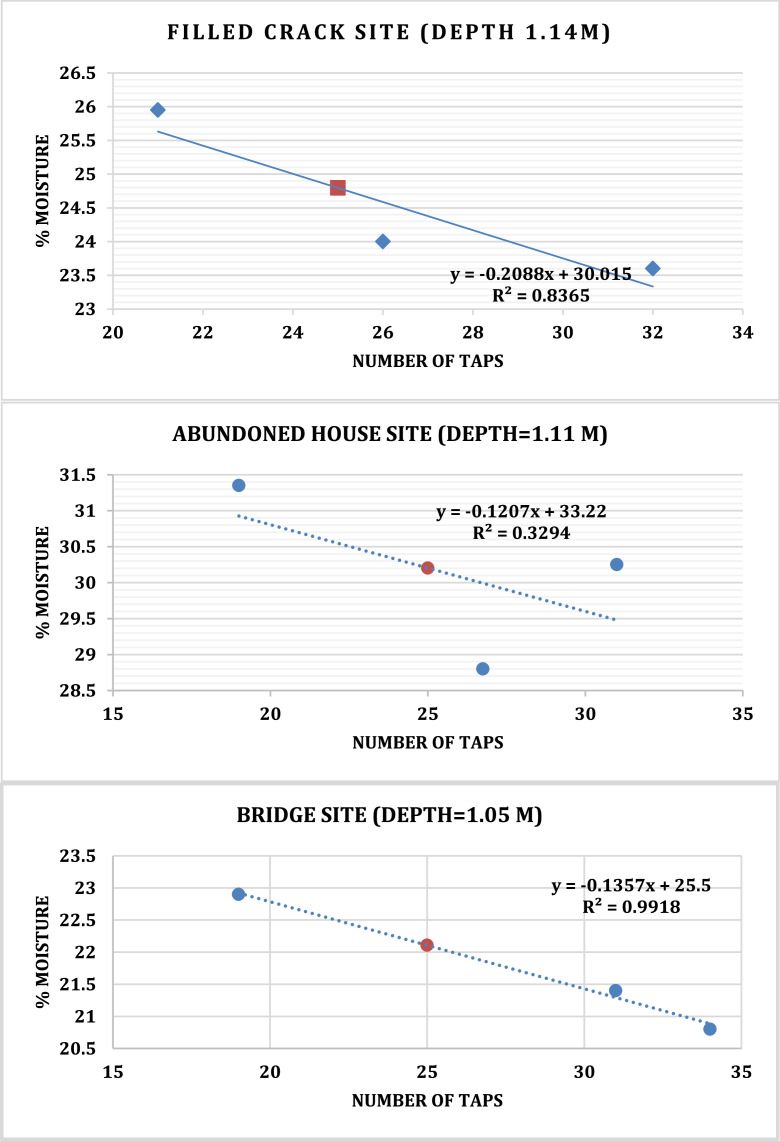
Liquid limit graph for soil samples corresponding to the 25th tap using the Cassagrande tool is found on the line of best fit to be 24.8 % for Filled Crack site, 30.20% for Abandoned House and 22.1% for the Bridge site.

The subdirectory named Soil test dataset has an excel file which shows plot of Atterberg limits. Soil analysis involved sieve analysis to determine the grain size distribution of soils that are greater than 0.075 mm in diameter [7]. The soil samples were placed into the top sieve and covered with a lid, and the whole stack was shaken for about 10 minutes. The weights of soil material trapped on each sieve and in the pan were then measured and tabulated as shown in Table 2. Liquid and plastic limit of soil samples are shown in Table 3 and associated plots for liquid limit vs number of taps are shown in Fig. 11. The Atterberg limits measured at the three survey sites indicate that the soil plastic index from depths of 1m falls within the range of 10-20% (medium plastic). The survey conducted at the Filled crack site shows a plastic index of 16%, while the Abandoned House and Bridge sites have 18.7% and 13.5%, respectively. The presence of clay content in soil is responsible for its plasticity, and there is a direct correlation between the degree of plasticity and the soil's potential for expansion. The Atterberg tests used to measure soil plasticity were the plastic and liquid limits tests. The soil samples obtained from the Filled Crack and Abandoned House sites both exhibited linear shrinkage of 6.4%, while the soil sample from the Bridge site showed a linear shrinkage of 2.9%

**Table 2:** Sieve analysis of soil samples collected from the three sites.

Sieve size	Filled Crack Site: (Initial soil sample mass: 1020 g, sample depth: 1.14 m)			Abandoned House site (Initial soil sample mass: 1000 g Soil sample depth: 1.11 m)			Bridge site (Initial soil sample mass: 1032 g, Sample depth: 1.05 m)		
	Mass retained	% Retained	%Passing	Mass retained	% Retained	%Passing	Mass retained	% Retained	%Passing
			99.9			99.9			100
2	46	4.5	95.4	35	3.5	96.4	42	4.1	95.9
1.18	112	11	84.4	125	12.5	83.9	129	12.6	83.3
0.6	271	26.6	57.8	259	26.0	57.9	204	19.9	63.4
0.425	123	12.1	45.7	142	14.2	43.7	150	14.6	48.8
0.3	109	10.7	35	146	14.6	29.1	162	15.8	33
0.15	236	23.2	11.8	213	21.3	7.8	253	24.6	8.4
0.075	82	8.1	3.7	59	5.9	1.9	66	6.4	2
Pan	38	3.7		19	1.9		21	2	
Total	1017 g	99.9 %		998 g	99.9 %		1027 g	100 %	

% retained = (individual mass retained/ total mass retained) *100; % passing = (total % retained – individual % retained).

**Table 3:** Liquid and plastic limit of soil samples collected from the three sites.

Container Number	*Filled Crack site (depth of 1.14 m)					**Abandoned House site: (depth of 1.11 m)					***Bridge site (depth of 1.05 m)				
	LIQUID LIMIT (%)			PLASTIC LIMIT (%)		LIQUID LIMIT (%)			PLASTIC LIMIT (%)		LIQUID LIMIT (%)			PLASTIC LIMIT (%)	
	6	1	4	5	8	2	8	5	4	7	5	8	2	7	2
Mass Cont& Wet Mat (m₂) (g)	61.22	60.90	61.60	53.96	51.93	59.56	60.70	60.39	55.93	59.10	63.92	61.68	63.43	69.17	67.16
Mass Cont + Dry Mat = m₃ (g)	57.26	57.24	57.44	52.98	51.15	55.32	56.49	56.13	54.29	57.26	59.84	58.19	59.70	67.0	65.3
Mass Container = m₁ (g)	41.98	41.97	39.80	41.99	41.88	41.80	41.88	42.0	39.82	41.81	41.99	41.88	41.79	41.82	41.80
Mass Moisture = (m₂ - m₃) (g)	3.96	3.66	4.16	0.98	0.78	4.24	4.21	4.26	1.64	1.84	4.08	3.49	3.73	2.17	1.86
Mass Dry Material = (m₃ - m₁) (g)	15.28	15.27	17.64	10.99	9.27	13.52	14.61	14.13	14.47	15.45	17.85	16.31	17.91	25.18	23.5
% Moisture =$(\frac{m_{2}-m_{3}}{m_{3}-m_{1}})*100\%$	25.9	24.0	23.6	8.91	8.41	31.4	28.8	30.15	11.3	11.9	22.9	21.4	20.8	8.62	7.91
Number of Taps	21	26	32	Mean	8.7	19	27	31	Mean	11.6	19	26	34	Mean	8.3

*LL (liquid limit) at the 25^th^ tap on the line of best fit is 24.8%. Other observed parameters are as follows: PL = 8.7%, Pi = 16%, LS = 6.4%.

**LL (liquid limit) at the 25^th^ tap on the line of best fit is 30.2 %. Other observed parameters are as follows: PL = 11.6%, Pi = 18.7%, LS = 6.4 %.

***LL (liquid limit) at the 25^th^ tap on the line of best fit is 22.1%, other observed parameters are as follows: PL = 8.3%, Pi = 13.5%, LS = 2.9%.

## 3 Experimental Design, Materials and Methods

The grid of Total Magnetic Intensity (TMI) used in this work was obtained from the Botswana Geoscience Institute. It was acquired in 1996 by the then Geological Survey of Botswana with a flight elevation of 80 m along north–south lines with a spacing of 250 m. The tie lines were oriented in the east–west direction and spaced 1.25 km apart [4]. The IGRF model of the core field was subtracted from the observed total field in order to obtain the residual total field. The minimum curvature technique [3] was used for gridding with a grid cell size of 62.5 m. The Geosoft Oasis Montaj package was used for aeromagnetic data processing [2].

Multi-channel Analysis of Surface Waves (MASW) is a non-invasive technique used to approximate the shear-wave velocity profile by utilizing the dispersive properties of Rayleigh waves to image subsurface layers. MASW surveys can be divided into active and passive surveys. In the active MASW method, surface waves are produced by an impulsive source such as a hammer, sledgehammer, weight drops, accelerated weight drops, or explosives. The passive MASW method, on the other hand, utilizes surface waves generated by natural sources or cultural activities [5]. The Multichannel Analysis of Surface Wave (MASW) method adopted in this work has significant advantages over other surface wave techniques, as all seismic wave energy, consisting of both body and surface waves, is recorded by multichannel receivers [8]. Seismic waves propagate in the form of body waves and surface waves. The difference between the two is that body waves are usually non-dispersive. In a solid and homogeneous medium, the velocity of surface waves does not fluctuate significantly as a function of distance propagated. However, when the properties of the medium vary with depth, surface waves become dispersive such that the velocity of propagation varies with respect to wavelength or frequency. The Multichannel Analysis of Surface Wave (MASW) method has an investigation depth shallower than 30 m. For this survey, geophones were arranged in a straight, equally spaced line on the surface of the test site across the fissure. The geophone spacing was 2 m, and the length of the spread was 46 m with 24 geophones. The offset distance was set at 4 m, which is the distance from the trigger to the first geophone. Elastic waves were generated using a sledgehammer at one end of the line, and the subsequent wave motion was recorded by the geophones as a function of time. A single shot gather was sufficient for 1D analysis of MASW [5]. The spread was shifted by 4 m, and a wave was generated, and this process continued until a reasonable length over the crack was covered. To investigate the fissured regions, three traverse profiles were selected. The first profile was identified across a gravel road within the village, running from one yard to another in an E-W direction. The fissure has since been filled up by villagers, and the coordinates of the profile at the beginning and end were recorded as -24.1410085°S 25.5872383°E and -24.1410867°S 25.586429°E, respectively. The second survey point was a fissure across a house (pink house), oriented SE-NW, causing the occupants to vacate the house. The survey line coordinates at the start and end of the spread were recorded as -24.1305751800°S 25.5917987823°E and -24.129640579°S 25.59136772°E respectively. The final chosen survey point was a fissure near the Bridge/tarred road. The ground crack stretches for more than 500 m towards Botlhapatlou village and also branches into a stream towards its endpoint. The survey line coordinates at the start and end of the profile were recorded as -24.12988°S 25.59150°E and -24.12992°S 25.58883°E, respectively. The average elevation of the three profile was measured to be 1040±10 m. The MASW profiles were run across the selected fissured areas, and the field configurations were set with an offset distance of 4 m and geophone spacing of 2 m. The sampling rate and record length were set at 0.25 ms and 1 ms, respectively. The spread was moved by 4 m until the whole survey line was covered. This involved moving the first two geophones to the end of the spread line, and three stacks were summed up per shot. Refer to Fig. 6 for more details.

To determine the percentage of different grain sizes within soil samples, a test was conducted. The test apparatus consisted of standard sieves of various openings, a pan, cleaning brushes, and a scale/balance. To carry out the procedure, the soil samples were first weighed, and the sieves were assembled in ascending order by placing those with large openings on top, followed by those with small openings, and finally, a pan at the bottom. The soil samples were then placed into the top sieve and covered with a lid. The next step involved shaking the sieve stack for a period of 10 minutes. The weights of soil material trapped on each sieve and in the pan were then measured, as shown in the Table 2. For the shrinkage limit test, the apparatus included mixing dishes, distilled water, spatulas, troughs, sample splitters, grease oil, and a drying oven. The soil samples were mixed with distilled water and left to absorb the water. Troughs (shrinkage dishes) were then prepared and greased before they were filled with the test samples. The soil was placed such that it took the level and shape of the troughs while preventing the formation of air spaces between the soil particles. The drying process then took place as the troughs containing the soil samples were placed inside an oven and left overnight at a temperature of 105°C. The presence of clay in the soil sample was noticed through a linear shrinkage of the sample during the drying process.

The liquid limit test was carried out using the Casagrande cup method. The apparatus used included the Casagrande liquid device, moisture cans, a spatula, a grooving tool, and a drying oven. The soil paste was placed in the Casagrande cup, and a groove was cut at the center of the soil paste with a standard grooving tool. The cup was then lifted and dropped from a height of 10 mm, during which the groove gradually closed up as a consequence of the lifting and dropping impact. Once the groove closed, the number of blows required for the groove to close was recorded, and the wet sample's mass, together with the moisture tin, was determined. The procedure was then repeated for the same sample at varying moisture contents. The samples were then placed in the oven and left overnight. Following the procedure, the moisture content lost during oven drying and the weight of the dry samples were determined. Lastly, the number of drops against the moisture content lost for each test was plotted on a graph, and the estimated water content corresponding to 25 blows was referred to as the liquid limit value. Plastic limit: The test was carried out using the following apparatus: mixing dishes, spatula, measuring cans, distilled water bottle, glass rolling surface and a drying oven. The procedure firstly involves measuring and recording of empty cans masses (m₁). Small portions of the sample were then rolled on a glass surface until crumbling takes place. The crumbled samples were weighed together with the empty cans (mass of container and wet material = m₂) and later placed in a drying oven. The mass container and dry material (m₃) were later recorded after the samples were removed from the drying oven, the mass of the moisture was determined through subtraction of m₃ from m₂ (i.e. m₂-m₃). The mass of dry material was also obtained through subtraction of m₁ from m₃ (m₃-m₁) and lastly the percentage of moisture was determined using the mass moisture and mass of dry material. The mean value of the moisture percentages gives the plastic limit value.

## Ethics Statements

This work is based on geophysical data acquired in the field and did not involve the use of human subjects, animal experiments or data collected from social media platforms.

## CRediT authorship contribution statement

**Olorato Mosweu:** Conceptualization, Methodology, Data curation, Writing – original draft, Writing – review & editing. **Moikwathai Moidaki:** Conceptualization, Methodology, Investigation. **Rubeni T. Ranganai:** Data curation, Writing – review & editing. **Lucky C. Moffat:** Data curation, Writing – review & editing. **Rapelang E. Simon:** Data curation, Writing – review & editing. **Kebabonye Laletsang:** Data curation, Writing – review & editing. **James G. King:** Data curation, Writing – review & editing. **Nata T. Tafesse:** Conceptualization. **Reneilwe Lasarwe:** Data curation. **Motsamai Kwadiba:** Data curation. **Mpho Ramaselaga:** . **Mooketsi Vike:** .

## Declaration of Competing Interest

The authors declare that they have no known competing financial interests or personal relationships that could have appeared to influence the work reported in this paper.

## Data Availability

Geo-technical Investigation of Soil Properties in Hatsalatladi Village, Botswana (Original data) (Mendeley Data).
